# Facile Synthesis of Multifunctional Carbon Dots from Spent Gromwell Roots and Their Application as Coating Agents

**DOI:** 10.3390/foods12112165

**Published:** 2023-05-27

**Authors:** Tanima Bhattacharya, Hyeon A. Do, Jong-Whan Rhim, Gye Hwa Shin, Jun Tae Kim

**Affiliations:** 1 Department of Food and Nutrition, Kyung Hee University, Seoul 02447, Republic of Korea; btanima1987@gmail.com (T.B.); angeia1003@naver.com (H.A.D.); jwrhim@khu.ac.kr (J.-W.R.); 2 BioNanocomposite Research Center, Kyung Hee University, Seoul 02447, Republic of Korea; 3 Department of Food and Nutrition, Kunsan National University, Gunsan 54150, Republic of Korea; winnie19@kunsan.ac.kr

**Keywords:** gromwell roots, carbon dots, antioxidant, edible coating, dye degradation

## Abstract

Spent Gromwell root-based multifunctional carbon dots (g-CDs) and sulfur-functionalized g-CDs (g-SCDs) were synthesized using a hydrothermal method. The mean particle size of g-CDs was confirmed to be 9.1 nm by TEM (transmission electron microscopy) analysis. The zeta potentials of g-CDs and g-SCDs were mostly negative with a value of −12.5 mV, indicating their stability in colloidal dispersion. Antioxidant activities were 76.9 ± 1.6% and 58.9 ± 0.8% for g-CDs, and 99.0 ± 0.1% and 62.5 ± 0.5% for g-SCDs by 2,2′-Azino-bis(3-ethylbenzothiazoline-6-sulfonic acid) (ABTS) and 2,2-diphenyl-1-picrylhydrazyl (DPPH) radical scavenging tests, respectively. In addition, the bathochromic shift of g-CDs is observed when their emission peaks appear at a higher wavelength than the excitation peaks. The prepared g-CDs and g-SCDs solutions were used as a coating agent for potato slices. The browning index of the control potato slices increased significantly from 5.0 to 33.5% during 24 to 72 h storage. However, the sample potato slices coated with g-CDs or g-SCDs suppressed the increase in the browning index. In particular, the browning index of the potato slices coated with g-SCDs ranged from 1.4 to 5.5%, whereas the potato slices coated with g-CDs had a browning index ranging from 3.5 to 26.1%. The g-SCDs were more effective in delaying oxidation or browning in foods. The g-CDs and g-SCDs also played a catalytic role in the Rhodamine B dye degradation activity. This activity will be useful in the future to break down toxins and adulterants in food commodities.

## 1. Introduction

Carbon quantum dots or carbon dots (CDs), a new emerging star in the carbon family, have gotten much attention because of their outstanding and tunable photoluminescence (PL), high quantum yield (QY), low cytotoxicity, small size, good biocompatibility, abundant, and low-cost. CDs have been applied in various fields, such as biomedical science, catalysis, optoelectronic devices, and anti-counterfeiting [1,2]. Biomass, particularly waste biomass, has numerous advantages over molecular carbon sources [3]. Many forms of biomasses, such as walnut peel [4], papaya [5], goose feather [6], bee pollen [7], and hibiscus [8], have been used as carbon sources to synthesize CDs in recent researches, and they have been used to various fields. Both carbonaceous core and surface functional groups give CDs their complicated chemical structure [9]. The synthesis process and the carbon sources used as raw materials affect the basic structure of CDs.

On the other hand, surface functional groups are often produced from a carbon source. The characteristics of CDs are controlled by their core and surface groups. For instance, the toxicity of CDs is determined by the former and how the latter disperses or interacts with other substances. However, CDs’ core and surface groups can be modified to improve their functionality during or after synthesis. The carboxyl, amino, hydroxyl, and other functional groups found in CDs are generally abundant and affect the surface properties of the CDs [10]. Surface passivating CDs with different chemicals can alter these surface functional groups. The development of applications in biological and imaging technologies is aided by surface functionalization, which increases the fluorescence production of CDs [11]. However, adding specific functional groups to surfaces also affects how CDs interact with biological surfaces and other crucial molecules found in living things, including DNA. For instance, the positive surface potential of polyethyleneimine (PEI)-modified CDs enables them to adsorb negatively charged DNA for applications involving gene transfer. Folate-modified N-doped CDs were specifically endocytic tumor cells with a specific cellular uptake of 93.40% [12], which further supports the use of surface-modified CDs in tumor therapy. Another investigation demonstrated CDs’ strong antimicrobial action. It exhibits remarkable antibacterial activity against *E. coli* when combined with other functional groups, including CDs with amine surface groups [13].

The emission spectra of CDs can be modified by doping with certain elements such as nitrogen (N), sulfur (S), phosphorus (P), boron (B), or their mixtures. Heteroatom functionalization is the most used technique for changing CDs. Reactants containing heteroatoms such as nitrogen, sulfur, and boron make it simple to incorporate them into CDs [14]. This CD functionalization with heteroatoms lessens toxicity and enhances functional qualities, including antioxidant and antibacterial capabilities. There have been claims that heteroatomic CDs are a more effective electrocatalyst for oxygen reduction than homoatomic CDs [15,16]. Additionally, heteroatomic CDs are believed to have a stronger bactericidal action.

Current research aims to create an environmentally friendly “green” method to manufacture CDs from natural precursors of medicinal plant Gromwell root biowastes. Gromwell root (*Lithospermum erythrorhizon*) has been used for dermatitis, autoimmune illnesses, neuroprotective effects, and chemotherapeutics [17]. Besides biomedical applications, only very few reports are available on active food packaging applications, such as pH indicator films with the shikonin colorant extracted from Gromwell root [18,19]. Shikonin-added cellulose nanofibers (CNF) film exhibited unique color changes depending on pHs ranging from 2 to 12 and improved mechanical strength and UV blocking capability without any sacrificing other properties such as transparency and thermal stability [20]. In this current study, spent Gromwell root, which is a waste of Gromwell root by shikonin extraction, was used to make CDs as a new functional material. As per our knowledge, there is no report on the study of plant root biowaste-based CDs. This study synthesized g-CDs and g-SCDs using a hydrothermal method using spent Gromwell root. Their functional properties were investigated by measuring antioxidant, antibacterial, and dye degradation activities. Our motivation is to develop active agents such as antibrowning or antimicrobial agents for food applications using natural precursor biowaste-based carbon dots.

## 2. Materials and Methods

### 2.1. Materials

Dried Gromwell roots (*Lithospermum erythrorhizon*) were purchased from the local market in Seoul, Republic of Korea. Moreover, 2,2′-Azino-bis(3-ethylbenzothiazoline-6-sulfonic acid) (ABTS), 2,2-diphenyl-1-picrylhydrazyl (DPPH), and potassium persulfate were purchased from Sigma-Aldrich (St. Louis, MO, USA). *Listeria monocytogenes* (ATCC 15313) and *Escherichia coli* O157: H7 (ATCC 43895) were acquired from the Korean Collection for Type Culture (KCTC, Seoul, Republic of Korea). Spent Gromwell root was obtained as a byproduct from the extraction process of shikonin, which is the functional colorant in Gromwell root. Shikonin was first extracted from Gromwell root powder using ethanol as following the previous study [18]. Gromwell roots were first processed into a fine powder using a blender. Further, 100 g of pulverized Gromwell powder was combined with 500 mL of 95% ethanol, gently mixed for one hour in the dark, and then subjected to one hour of sonication using an ultrasonicator with a water bath (FS 140H, Ultrasonic Cleaner, Fisher Scientific, Pittsburg, PA, USA). The brilliant red extracted solution was concentrated at 50 °C using a rotary vacuum evaporator, filtered to remove insoluble components, freeze-dried to produce the shikonin powder, and then kept in the dark at 4 °C until needed. The residue after extraction of shikonin is dried at room temperature to obtain spent Gromwell root powder.

### 2.2. Synthesis of g-CDs and g-SCDs

**Figure 1:**
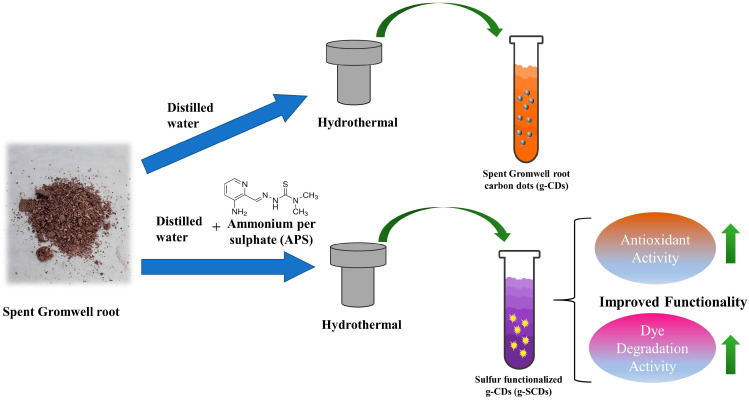
Schematic diagram of g-CDs and g-SCDs synthesis.

In a one-pot hydrothermal technique, spent Gromwell root was used to prepare carbon quantum dots (CDs). Dried spent Gromwell root (1 g) was added to 50 mL of distilled water in a Teflon-lined stainless-steel reactor (100 mL) and heated in an oil bath with varying temperatures at 200–250 °C for 6 h. After cooling, the solid part was separated by centrifugation at 8000 rpm for 30 min. The recovered supernatant was filtered via a Whatman membrane filter with a 25 mm diameter and pore size of 0.22 μm (Whatman International Ltd., Maidstone, UK) to remove the impurities. Sulfur-doped g-CDs (g-SCDs) were simultaneously prepared by adding 0.1 g of ammonium persulfate before hydrothermal synthesis at 200 °C for 6 h. The pristine g-CDs formed at 200 °C were used to prepare the g-SCDs because they had better optical properties than the ones formed at 225 °C and 250 °C. The recovered supernatant was filtered using a Whatman membrane filter to remove the impurities (Figure 1).

### 2.3. Optical and Physicochemical Properties

A morphological study, including the size and shape of g-CDs, was carried out using a transmission electron microscope (TEM, Tecnai F20 G2, FEI Co., Hillsboro, OR, USA). Zeta potential was analyzed using a zeta potential analyzer (ELSZ-2000, Otsuka Electronics Co., Osaka, Japan). The light absorbance of the g-CDs was measured using a UV-Vis spectrophotometer (Mecasys Optizen POP Series UV/Vis, Seoul, Republic of Korea) with a wavelength range of 200–800 nm. Fluorescence of the g-CDs and g-SCDs was measured using a fluorescence spectrophotometer equipped with a quartz cuvette (FluoroMax, Horiba, Piscataway, NJ, USA). The emission intensity was adjusted for excitation power to compare intensities. Excitation increments of 20 nm were used for the excitation-emission scans.

### 2.4. Antioxidant Activity of g-CDs and g-SCDs

The antioxidant activity of g-CDs and g-SCDs at various concentrations (25, 50, 75, and 100 μg/mL) was evaluated using 2,2′-azino-bis (3-ethylbenzothiazoline-6-sulfonic acid) (ABTS) and 2,2-diphenyl-1-picrylhydrazyl (DPPH) radical scavenging assays. For the ABTS assay, ABTS solution (7 mM) and potassium persulfate (2.45 mM) was mixed in a ratio of 1:1 and incubated for 16 h. The mixture was diluted with distilled water to produce an absorbance of 1.00 at 734 nm. For the DPPH assay, 4 mg of DPPH was dissolved in 100 mL of methanol. The different concentrations of g-CDs and g-SCDs were added to the 10 mL of ABTS and DPPH solution. The reaction was conducted at room temperature in the dark for 30 min, and the absorbance was measured at 734 nm and 517 nm, respectively. The radical scavenging activity was calculated using the following equation:

Radical scavenging activity (%) = (A_c_ − A_s_)/A_c_ × 100 (1)

where A_c_ and A_s_ were the absorbances of ABTS and DPPH solutions of the control and test solutions, respectively.

### 2.5. Antibacterial Activity of g-CDs and g-SCDs

The antibacterial activity of the g-CDs and the g-SCDs was evaluated using the well-diffusion method against *Listeria monocytogenes*. Six Log CFU/mL of bacterial broth was applied on the surface of TSB/BHI plates using a sterilized swab. Then, 8 mm-diameter wells were punched on agar, filled with 100 µL of g-CDs and g-SCDs (6.4 mg/mL), and incubated at 37 °C for 24 h. Then, the diameter of the inhibitory zone was determined.

### 2.6. Catalytic Activity

The catalytic activity of g-CDs and g-SCDs was assessed for the degradation of Rhodamine-B (Rh-B) in the presence of sodium borohydride (NaBH4) using a UV-Vis spectrophotometer [21]. The concentration of the reactants and catalyst was determined using the modified procedure [21]. A 3 mL complete solution was created by filling two cuvettes with 0.2 mL of freshly made NaBH_4_ (0.50 M), 1 mL of Rh-B (0.01 M), and 1.8 mL of water. Then, 20 µL (0.2 mg/mL) of the CD samples were introduced to one of the cuvettes mentioned above, while the other acted as the control. The degradation of Rh-B was investigated using UV-visible spectroscopy to record the time-dependent absorbance of the reaction mixture at 554 nm wavelength every two min.

Degradation (%) = (A_0_ − A_t_)/A_0_ × 100 (2)

where A_t_ denotes the absorbance at the reaction time (t) and A_0_ denotes the initial absorbance at t = 0.

### 2.7. Visual Appearance and Image Analysis of Potato Slices

Browning of potato slices after coating with g-CDs and g-SCDs solutions was observed for 72 h. The sliced potatoes were dipped in g-CDs and g-SCDs solutions, and dried at room temperature. The dipping and drying process was repeated three times to make a complete coating layer. The control was prepared by dipping and drying the sliced potatoes in distilled water three times. The percentage of browning over storage time was analyzed by Image J software, slightly modifying the work of Carpentier et al. [22] and Lunadei et al. [23]. This method uses the distribution of the image histogram to calculate the threshold level. In our case, the pictures of the coated potato slices were captured and analyzed offline in Image J software. Initial segmentation methods used to separate samples from the background included the Otsu approach.

### 2.8. Statistical Analysis

All measurements were made in triplicate using freshly made samples, and the results were presented as means and standard deviations (mean ± SD). One-way analysis of variance (ANOVA) was used to run statistics on a completely randomized design using SPSS software (SPSS Inc., Chicago, IL, USA). Duncan’s multiple range test (*p* < 0.05) was used to separate the difference between the test groups.

## 3. Results and Discussion

### 3.1. Optical Properties of g-CDs and g-SCDs

**Figure 2:**
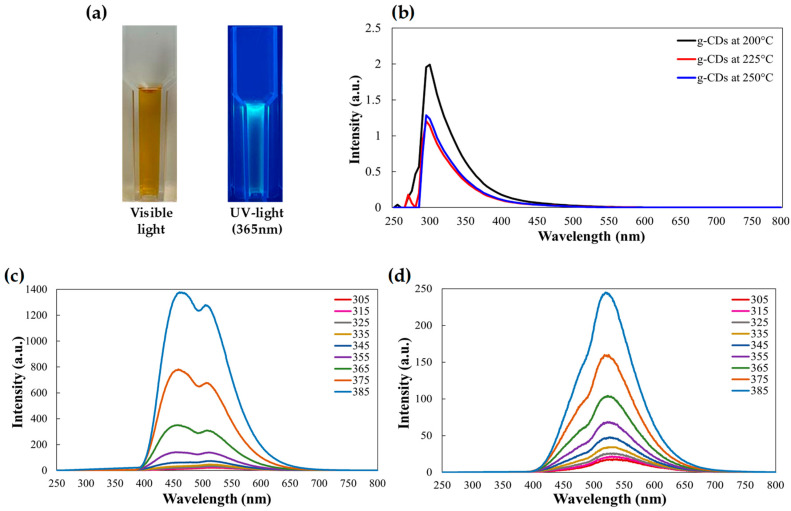
(**a**) The images of g-CDs in visible light and UV light at 365 nm, (**b**) UV spectra of g-CDs at different temperatures, (**c**) photoluminescence spectra of g-CDs, and (**d**) photoluminescence spectra of g-SCDs.

The g-CDs solution exhibits a yellowish-brown color that vividly changes to blue in ultraviolet light due to the quantum confinement effect, as shown in Figure 2a,b. The observed phenomena are comparable with previously reported carbon dots produced by curcumin precursor [24]. The high intensity and, consequently, a high amount of CDs at 200 °C were produced by the concentration of 1 g of the plant root sample in 50 mL of distilled water. Then, using a concentration of 1 g/50 mL, CD preparation was assessed at various calcination temperatures. At 200 °C, the maximum efficiency was attained. At greater temperatures, no increase in efficiency was seen.

Figure 2c shows the UV-Vis spectra of g-CDs prepared at 200 to 250 °C. The g-CDs prepared at 200 °C were chosen as their intensity was highest among the three g-CDs. UV-Vis spectra were utilized to observe the visual and optical characteristics of water-soluble g-CDs. Due to the n–* transition of CO or the C–OH bond, the g-CDs peak at about 290 nm, demonstrating the successful synthesis of carbon dots via carbonization of Gromwell root biowastes (Figure 2c). A prominent emission peak in the blue light area was visible in the photoluminescence spectra of the CD solution at the excitation wavelength, and the emission could be adjusted using an incoming excitation wavelength range of 350–650 nm (Figure 2c,d). As the excitation energy rose from 305 to 385 nm, the emission peak underwent a strong bathochromic (red) shift with reduced intensity. Surface imperfections, which provide tunable emissions with various energy levels, are one of the most plausible reasons for this coordinated emission. Photoluminescence emission peak was observed at 385 nm for the g-CDs. Whereas this may be due to high concentration, the photoluminescence is observed at low intensity in the case of g-SCDs.

### 3.2. TEM Images and Zeta Potential of g-CDs

**Figure 3:**
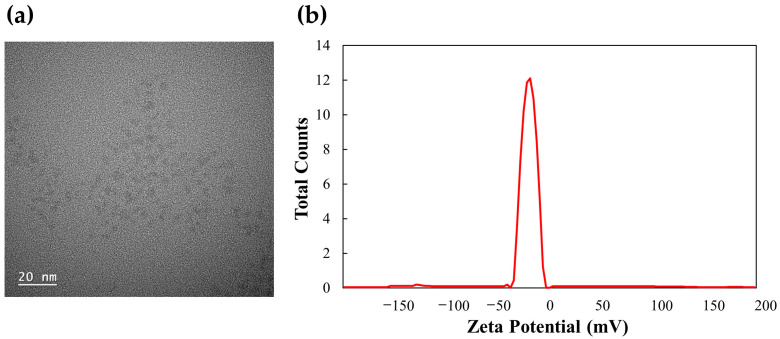
(**a**) TEM image and (**b**) zeta potential of g-CDs.

Figure 3a shows the TEM image of the g-CDs. The shape of the g-CDs was truncated triangular nanoparticles with diameters less than 10 nm. In some cases, particle sizes of CDs larger than 10 nm have been reported, which is attributed to the aggregation of CDs [24,25,26]. The cluster of CDs in the solid state was due to their small size. The g-CDs were mostly negatively charged and tended to attain stability in colloidal solution as their zeta potential of −12.5 mV, as shown in Figure 3b.

### 3.3. Antioxidant Activity of g-CDs and g-SCDs

**Figure 4:**
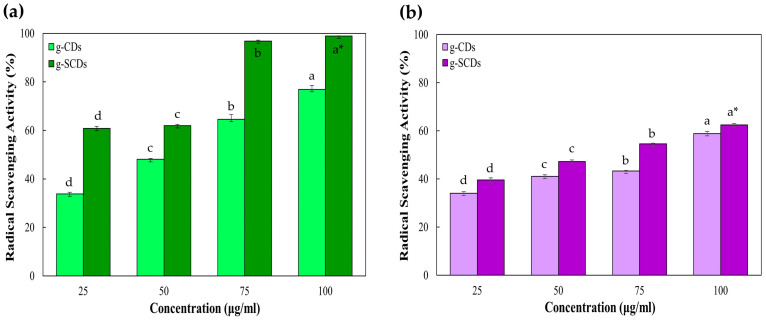
Antioxidant activity of g-CDs and g-SCDs by ABTS (**a**) and DPPH (**b**) radical scavenging assay. * Different letters in the same sample indicate a significant difference at *p* < 0.05 (*n* = 3).

Figure 4a,b show the antioxidant activity by ABTS and DPPH radical scavenging assay of the g-CDs and the g-SCDs as a function of concentration. The antioxidant activities of the g-CDs at 75 and 100 μg/mL concentrations were 64.6 ± 1.9~76.9 ± 1.6% and 43.4 ± 0.3~58.9 ± 0.5% by ABTS and DPPH assay, respectively. On the other hand, the antioxidant activities of the g-SCDs at 75 and 100 μg/mL concentrations were 96.8 ± 0.3~98.9 ± 0.1% and 54.6 ± 0.3~62.5 ± 0.5% by ABTS and DPPH assay, respectively. The antioxidant activity of the g-CDs and the g-SCDs was significantly increased with the concentrations of the g-CDs and the g-SCDs. ABTS radical scavenging activity of both CDs was higher than DPPH because the g-CDs are highly soluble in an aqueous ABTS solution than in a methanol DPPH solution. Stronger antioxidant activity in the ABTS approach is mostly due to hydrophilicity and greater g-CDs dispersion in an aqueous solution, which is consistent with the findings indicating decreased the free radicals scavenging capacity in the DPPH method. The reduced interaction of g-CDs and DPPH free radicals in methanol solution is the cause of the DPPH method’s lower antioxidant activity. The hydroxyl group of the surface functional group is believed to be the source of g-SCDs antioxidant activity. As the hydroxyl group is transformed into hydrogen ions (H^+^), it is known that ABTS and DPPH radicals are quenched. The production of more hydroxyl (OH^−^) radicals is the cause of the decreased free radical scavenging activity of the g-CDs compared to the g-SCDs. The ability to quench free radicals using ABTS or DPPH is prevented by stability restoration and binding of the radicals. The existence of surface functional groups on the g-CDs may explain their remarkable antioxidant activity, similar to Roy et al. [24]. The antioxidant activity of roots-based carbon dots has greater radical scavenging activity than other aerial parts of the plant. Nasseri and co-workers [27] observed a comparative study of carbon dots from various parts such as roots, flowers, and aerial parts of the medicinal plant *Echinops persicus*. Additionally, 50% radical scavenging activity was observed from the concentration of 6 g/75 mL of root-based CDs. Moualek et al. [28] observed that a methanol extract of *Arbutus unedo* could exhibit DPPH radical scavenging activity of nearly 30~40% at a concentration of 7.5 μg/mL. Therefore, g-CDs and g-SCDs were less effective than methanol extract of medicinal plants in radical scavenging activity, but superior to other CDs.

### 3.4. Antibacterial Activity of g-CDs and g-SCDs

**Figure 5:**
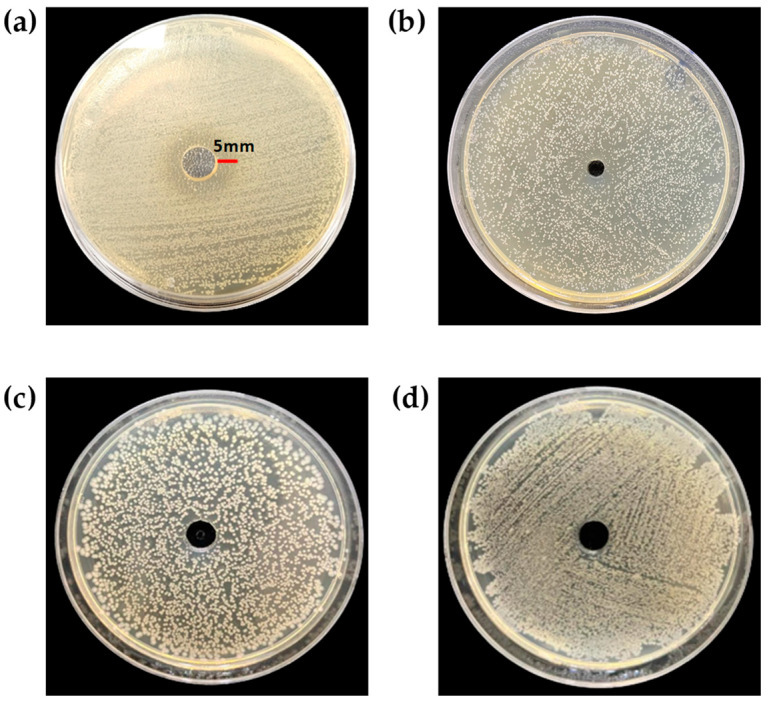
Antibacterial activity of g-CDs (**a**,**c**) and g-SCDs (**b**,**d**) against *L. monocytogenes* (**a**,**b**) and *E. coli* (**c**,**d**) by inhibition zone, respectively.

Figure 5 shows the antibacterial activity of the g-CDs and the g-SCDs against *Listeria monocytogenes* and *Escherichia coli* by the inhibition zone method. The g-CDs showed 20 mm and 0 mm inhibition zone against *L. monocytogenes* and *E. coli*. The g-SCDs did not show any inhibition zone. The inhibition zone is the observed circular patch marked on the Petri dish and no bacterial colonies have grown in that zone. This is an area of the medium where bacteria cannot thrive because there are carbon dots that hinder bacterial growth [18]. As most CDs are smaller than bacteria, they readily penetrate bacterial cells and internalize into cells, increasing oxidative capacity and triggering enzyme inhibition, causing cells to rupture and triggering mechanisms of oxidative stress leading to cell lysis, causing death [24]. Our results contradict Roy et al. [24] as their functionalized CDs were antibacterial compared to pristine carbon dots from turmeric (*Curcumin rhizhome*) precursors. The g-CDs exhibit excellent antibacterial action against bacterial strains may be due to their high singlet oxygen generation levels. The doping of the g-CDs with ammonium persulfate heteroatom decreases the antibacterial capability of these CDs. Compared to *E. coli*, *L. monocytogenes* was more sensitive to the g-CDs. This outcome might result from the more complicated structure of *E. coli*. Cytoplasmic membrane, peptidoglycan, and outer membrane comprise its three envelope layers. This may prevent weak singlet oxygen-potent g-CDs or g-SCDs from degrading the *E. coli* membranes. A similar report is observed in the case of fluorine-doped and chlorine-doped CDs, which failed to show promising antibacterial effects may be due to reduced reactive oxygen species [26]. The antibacterial efficacy of methanolic leaf extract was reported in a study [29] against various microorganisms including *Pseudomonas aeruginosa*, *Escherichia coli*, *Klebsiella pneumoniae*, *Salmonella typhimurium*, and *Staphylococcus aureus*. The extract showed the most significant inhibition zone at 160 mg/mL concentration against *E. coli* (19 mm), *P. aeruginosa* (18 mm), and *K. pneumoniae* (17 mm). Therefore, it can be said that the g-CDs and g-SCDs of this study showed improved antibacterial efficacy against bacteria even at a low concentration of 6 mg/mL.

### 3.5. Rhodamine Dye Degradation Activity of g-CDs and g-SCDs

**Figure 6:**
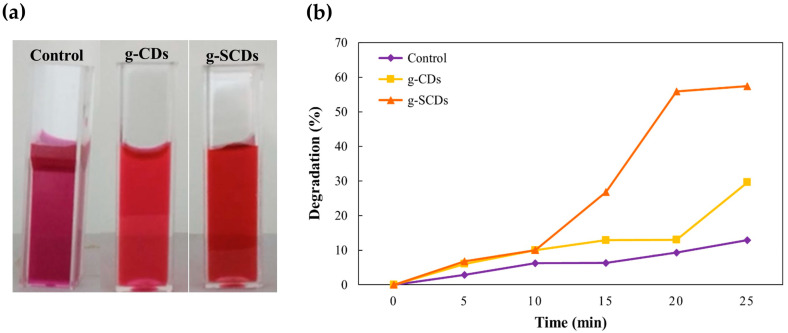
Visual appearance of Ph-B dye color change (**a**) and (**b**) dye degradation percentage of control, g-CDs, and g-SCDs.

The g-SCDs result in an approximately 57.4% quicker degradation of Rhodamine-B dye after 25 min, as shown in Figure 6. The catalytic effect is due to its tiny size and high surface volume ratio, making it an effective dye degradation substrate. The oxidation of aqueous sodium borohydride solution with a concentration of the g-CDs and g-SCDs as catalysts liberates hydrogen bubbles in the reaction mechanism. Metaboric ions (BO_2_^−^) and protonated hydrogen atoms are formed when borate ions (BH_4_) adsorb on the surface of catalysts oxidized with water molecules. Due to its nucleophilic and electrophilic natures, the catalyst reduces the reductive potential of NaBH_4_ while increasing the reductive potential of Rh-B dye. Furthermore, the Rh-B dye and borate ion (BH_4_) is attributed to the adsorption of the g-CDs and the g-SCDs, serving as a mediator (electron relay system) to accept an electron from borate ions and transfer it to Rh-B via its surface. During this reduction process, constant H_2_ gas bubbles are produced, breaking the central core in the molecule and lowering the barrier between reactants and the products. The pink colors of Rh-B faded to lighter as the dye’s chromophore structure was broken down into less harmful forms. As a result, the vast interfacial ratio due to the reduced size and the efficient electron-accepting capability owing to sulfur doping could cause the high catalytic activity of the g-CDs. The reaction mechanism is somewhat similar to Pandey et al. [30]. Figure 6a,b represent the visual appearance of color change and dye degradation percentage of Rh-B dye by the g-CDs and the g-SCDs. The control used in the experiment is the dye itself without incorporating any additive such as pristine functionalized CDs. Despite limited reports on medicinal plant-derived carbon dots and their dye degradation applications, Mejing et al. [31] synthesized carbon dots from the Chinese herbal *Alisma rhizome*. The researchers observed CDs’ photodegradation catalytic activity of malachite green dye with a concentration of 0.6 mg/mL over time. The photodegradation activity of the CDs was 76% after 1 h, and increased to 88 to 100% after 2 h and 4.5 h, respectively [27]. They used a high volume of CDs (2 mL) and a longer time to show good photodegradation activity. However, in this study, 57.4% photodegradation activity was obtained after 25 min using 0.2 μL of CDs. It can be assumed that our CDs catalytic activity is better in response to Mejing et al. [31]. Therefore, g-SCDs can be used to degrade toxic substances or additives to achieve the purpose of detecting contaminants in food.

### 3.6. Visual Appearance of Potato Slice after Coating with g-CDs and g-SCDs

**Figure 7:**
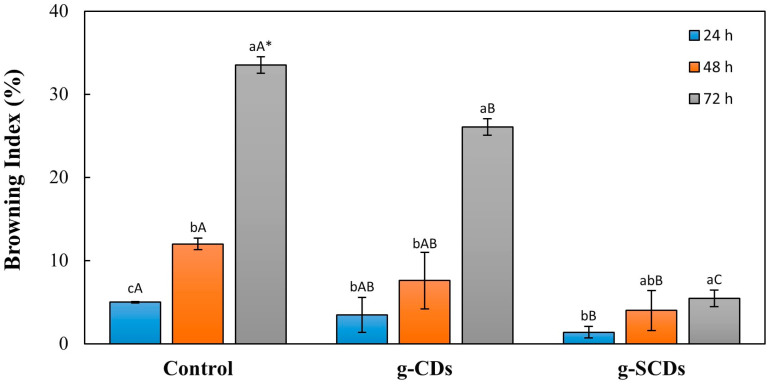
Browning index of potatoes dipped in distilled water (control), g-CDs, and g-SCDs at 24, 48, and 72 h. * Uppercase and lowercase letters indicate a significant difference at *p* < 0.05 (*n* = 3) by the samples and time, respectively.

**Figure 8:**
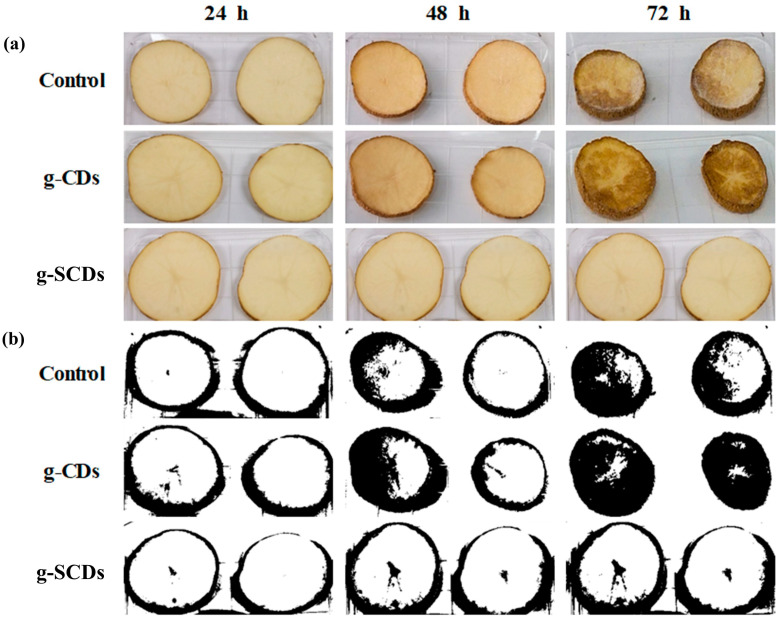
(**a**) Appearance images and (**b**) image analysis by Image J software of potatoes dipped in distilled water (control), g-CDs, and g-SCDs at 24, 48, and 72 h.

The browning index of the potato slices after coating with distilled water (control), g-CDs, and g-SCDs was compared at intervals of 24, 48, and 72 h (Figure 7). The browning index for the white part of the image was calculated, the average value was obtained, and the standard deviation was calculated using Image J software. The browning index of the control was significantly increased from 5.0 to 33.5% at 24 h to 72 h storage. However, coating with the g-CDs and g-SCDs retarded the increasing browning index of potato slices. The potato slices coated with g-CDs ranged the browning index from 3.5 to 26.1%, whereas the sample coated with g-SCDs ranged from 1.4 to 5.5%. All these findings supported the potential of the suggested method for classifying fresh-cut potatoes according to their state of browning. This method could be a potential criterion for determining the shelf life for fresh-cut potato slices stored at room temperature with or without additional inhibitory treatments. Thus, the image analysis results also tally with the antioxidant mechanism, which denotes the g-SCDs’ exceptional antioxidant ability by their power to scavenge free radicals. Figure 8a,b represent the camera and analytical images of the coated potato slices over storage time using Image J software, respectively.

To the best of our knowledge, there are limited or no reports of carbon dots as anti-browning agents. Researchers in the current study found several established reports of applying nanocoatings to extend the shelf life of cut fruits and vegetables. Gvozdenko et al. [32] synthesized copper oxide (CuO) nanoparticles and observed their efficacy in the storage of strawberries and tomatoes. During the experiment, they observed that untreated control samples of strawberries showed signs of rotting on day 4, whereas tomatoes started to rot on day 7. However, no corrosion or discoloration was observed in the samples treated with CuO nanoparticles. In another study by Zambrano-Zaragoza et al. [33], freshly cut apples were dip-coated with xanthan gum, nanoemulsion, nanocapsule, nanosphere, and their mixture. There was no significant difference in the browning index between xanthan gum and xanthan gum/nanoemulsion, while the sample coated with nanosphere/nanoemulsion showed a potential in lowering the browning index. The results indicate that the submicron-size coating agent helps extend the product’s shelf life compared to the control and xanthan gum coating. In this study, g-CDs-coated potatoes and g-SCDs-coated potatoes showed no fungal or bacterial growth during storage at 25 °C for 72 h. Only the surface was dry and the potatoes lost some moisture over time and became soggy after 72 h.

## 4. Conclusions

CDs were prepared with 1 g/50 mL of spent gromwell root powder extract at 200 °C using a hydrothermal method. The g-CDs have an average diameter of 9.1 nm, with a surface charge of −12.5 mV. The g-CDs also exhibited a bathochromic shift, indicating a redshift confirming the formation of CDs. The g-CDs showed high efficacy in radical scavenging activities when promising antioxidant properties were revealed in DPPH and ABTS assay. This antioxidant characteristic can be utilized in the food industry to inhibit or delay browning in cut vegetables, even at room temperature. The browning index of potato slices coated with g-CDs ranged from 3.5 to 26.1%, whereas the sample coated with g-SCDs ranged from 1.4 to 5.5%. The sulfur-functionalized g-CDs (g-SCDs) enhanced antioxidant activity in DPPH and ABTS assay. ABTS and DPPH radical scavenging activities of the g-SCDs at 100 µg/mL were 98.9 ± 0.1% and 62.5 ± 0.5%, respectively. It was observed that the g-SCDs improved the catalytic activity of g-CDs, which will effectively determine toxicants and additive colorants, dyes, and pesticides. Thus, the g-CDs and g-SCDs can be used as fillers for bioactive food packaging, extending the shelf life and quality of food materials.

## Data Availability

Data is contained within the article.
